# Bioassay and molecular monitoring of insecticide resistance status in *Aedes albopictus* populations from Greece, to support evidence-based vector control

**DOI:** 10.1186/s13071-020-04204-0

**Published:** 2020-06-29

**Authors:** Sofia Balaska, Emmanouil A. Fotakis, Ilias Kioulos, Linda Grigoraki, Spyridoula Mpellou, Alexandra Chaskopoulou, John Vontas

**Affiliations:** 1 Department of Crop Science, Pesticide Science Lab, Agricultural University of Athens, Athens, Greece; 2 Institute of Molecular Biology and Biotechnology, Foundation for Research and Technology Hellas, Heraklion, Greece; 3 Department of Vector Biology, Liverpool School of Tropical Medicine, Pembroke Place, Liverpool, UK; 4 Bioefarmoges Eleftheriou LP -Integrated Mosquito Control, Marathon, 19007 Greece; 5 European Biological Control Laboratory, USDA-ARS, Thessaloniki, Greece

**Keywords:** Diagnostic, Bioassay, Arbovirus, Vector control, Mosquito tiger, Insecticide resistance, *kdr*, *Bti*, Gene amplification

## Abstract

**Background:**

*Aedes albopictus* has a well-established presence in southern European countries, associated with recent disease outbreaks (e.g. chikungunya). Development of insecticide resistance in the vector is a major concern as its control mainly relies on the use of biocides. Data on the species’ resistance status are essential for efficient and sustainable control. To date the insecticide resistance status of *Ae. albopictus* populations from Greece against major insecticides used in vector control remains largely unknown.

**Methods:**

We investigated the insecticide resistance status of 19 *Ae. albopictus* populations from 11 regions of Greece. Bioassays were performed against diflubenzuron (DFB), *Bacillus thuringiensis* var. *israelensis* (*Bti*), deltamethrin and malathion. Known insecticide resistance loci were molecularly analysed, i.e. voltage-gated sodium channel (*VGSC*) mutations associated with pyrethroid resistance; presence and frequency of carboxylesterases 3 (*CCEae3a*) and 6 (*CCEae6a*) gene amplification associated with organophosphate (OP) resistance and; chitin synthase-1 (*CHS-1*) for the possible presence of DFB resistance mutations.

**Results:**

Bioassays showed full susceptibility to DFB, *Bti* and deltamethrin, but resistance against the OP malathion (range of mortality: 55.30–91.40%). *VGSC* analysis revealed a widespread distribution of the mutations F1534C (in all populations, with allelic frequencies between 6.6–68.3%), and I1532T (in 6 populations; allelic frequencies below 22.70%), but absence of V1016G. *CCE* gene amplifications were recorded in 8 out of 11 populations (overall frequency: 33%). Co-presence of the F1534C mutation and *CCEae3a* amplification was reported in 39 of the 156 samples analysed by both assays. No mutations at the *CHS-1* I1043 locus were detected.

**Conclusions:**

The results indicate: (i) the suitability of larvicides DFB and *Bti* for *Ae. albopictus* control in Greece; (ii) possible incipient pyrethroid resistance due to the presence of *kdr* mutations; and (iii) possible reduced efficacy of OPs, in a scenario of re-introducing them for vector control. The study highlights the need for systematic resistance monitoring for developing and implementing appropriate evidence-based control programmes.
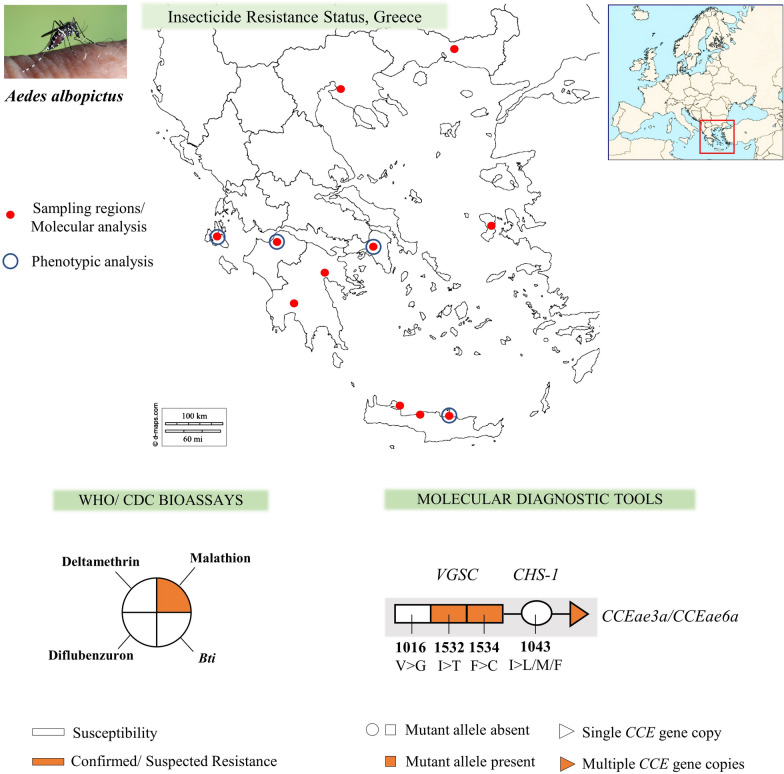

## Introduction

In the last two decades southern European countries have suffered from a number of vector-borne diseases (VBDs), such as chikungunya (CHIKV), dengue (DENV) and West Nile virus (WNV) outbreaks [1, 2]. This has been attributed to several factors including the introduction and establishment of invasive vector species, associated with the ongoing environmental and climate change and the globalization of human activities [3].

*Aedes albopictus* is an important vector and invasive mosquito species. Originating from the tropical and subtropical regions of southern Asia, it currently displays a worldwide distribution [4] facilitated by its ecological plasticity, competitive behavior and egg diapause/dormancy behaviour during the winter period [5]. *Aedes albopictus* was first recorded in Europe in 1979 [6] and to date displays a wide-spread distribution in the Mediterranean region while also reported in northern European countries [7]. Being a competent vector of more than 22 arboviruses, including CHIKV and DENV [8], *Ae. albopictus* constitutes a major public health threat. Its presence in Europe has been associated with the local transmission of CHIKV in Italy (2007 and 2017) and southeastern France (2010 and 2014) and of DENV in France (2010, 2013–2015), Spain (2014–2016) and Croatia (2010) [1]. Furthermore, since 2016 more than 2400 imported Zika cases have been reported in Europe [9, 10], which in conjunction with the known distribution of *Ae. albopictus* and Zika vectorial capacity raises concerns for potential autochthonous Zika transmission [11].

*Aedes albopictus* has also been reported in Greece [12], a country witnessing consecutive WNV outbreaks since 2010, with the largest outbreak in 2018 resulting in a total of 316 human cases and 50 fatalities [13]. Although *Culex pipiens* has been implicated as the primary WNV vector in Greece [14], *Ae. albopictus* could potentially have a role in disease transmission, as a secondary vector [5, 15]. Apart from a major public health concern, *Ae. albopictus* causes an important biting nuisance problem, negatively affecting the quality of life and potentially acting as a discouraging factor for the tourist economy [16].

The prevention of mosquito-borne diseases (MBDs) relies to a great extent on vector control [17] and the utilization of synthetic insecticides [18]. In Europe, pyrethroids (e.g. permethrin, deltamethrin and alpha-cypermethrin) and the larvicides diflubenzuron (DFB) and pyriproxyfen are the main insecticides used to control adult and immature stage *Aedes* mosquitoes, respectively [18, 19]. In Greece, applications of *Bacillus thuringiensis israelensis* (*Bti*) and of the insect growth regulator (IGR) DFB compose the majority of the vector control interventions implemented in large-scale (regional level) control programmes [20]. Pyrethroid insecticides, including type I (non-alpha-cyano group, such as permethrin) and type II (alpha-cyano group, such as deltamethrin and alpha-cypermethrin) formulations, are registered and used in professional vector control programmes and for personal protection/household level applications [21]. A major issue associated with the intensive use of a limited number of insecticides in mosquito and agricultural pest control is the development of insecticide resistance [18]. Insecticide resistance has been reported in *Ae. albopictus*, yet a big knowledge gap remains in regards to the susceptibility status, geographical distribution, frequency and co-occurrence of resistance traits and underlying mechanisms in the vector populations. Bioassay experiments have recorded resistance to several insecticides including pyrethroids, DDT, temephos, malathion, etc. mainly in rural and urban central and southern Asian populations (e.g. from China, Thailand, Singapore and India), while only sporadic cases of resistance have been reported in Europe, America and western Africa [18].

The two insecticide resistance mechanisms reported so far in *Aedes* mosquitoes are (i) target-site resistance, involving mutations at the insecticide’s target site of action and (ii) metabolic detoxification, obtained through overexpression or conformational changes of enzymes involved in the metabolism of the insecticide [18, 22]. To date, five mutations in two loci of the voltage-gated sodium channel (*VGSC*) gene; V1016G (valine to glycine), I1532T (isoleucine to threonine) and F1534C/L/S (phenylalanine to cysteine/leucine/serine), have been reported in *Ae. albopictus* populations from central and southeastern Asia, European Mediterranean countries, the USA and Brazil [18].

The involvement of these five mutations in insecticide resistance has been further examined by expressing the mutated *VGSC* channels in *Xenopus* eggs and investigating how their electrophysiological properties are affected in the presence of insecticides. All three mutations at position F1534 (F1534C/L and S), as well as the I1532T mutation have been shown to significantly reduce the channel’s sensitivity to type I but not to type II pyrethroids [23, 24]. The V1016G mutation also reduces the channel’s sensitivity mainly to permethrin and slightly to deltamethrin [23]. However, a synergistic effect has been reported in the presence of the triple mutant V1016G + F1534C + S989P (a third mutation identified in *Ae. aegypti* populations), which substantially reduced sensitivity to both permethrin and deltamethrin [25].

Pyrethroid resistance has also been correlated to *CYP6P12* over-expression conferring increased metabolic detoxification [26]. Metabolic resistance to the larvicide temephos (OP) has been functionally associated with the upregulation of carboxylesterases CCEae3a and CCEae6a, due to gene amplification [27]. *Aedes albopictus* populations from Greece and Florida (USA) have been found to carry this *CCEae3a* gene amplification or the *CCEae3a-CCEae6a* co-amplification [28]. Regarding alternative insecticides, such as IGRs or *Bti*, no genotypic resistance data have been reported for *Aedes* mosquitoes, but three point mutations I1043M/L/F in the chitin synthase-1 (*CHS-1*) gene were recently identified in *Cx. pipiens* mosquitoes, conferring very high levels of resistance to the larvicide DFB [29–31].

The re-appearance of VBDs in Europe, the widespread distribution of *Ae. albopictus* in southern Europe, the sporadic information on the vectors’ insecticide resistance status and the need for evidence-based mosquito control programmes acting in advance of disease outbreaks, make the monitoring and analysis of the *Ae. albopictus* insecticide resistance traits a necessity [32]. Here, we analysed the insecticide resistance status in a number of *Ae. albopictus* populations from Greece, using bioassays and molecular genotyping assays targeting known resistance markers.

## Methods

### Study localities, sample collections and mosquito handling

**Fig. 1:**
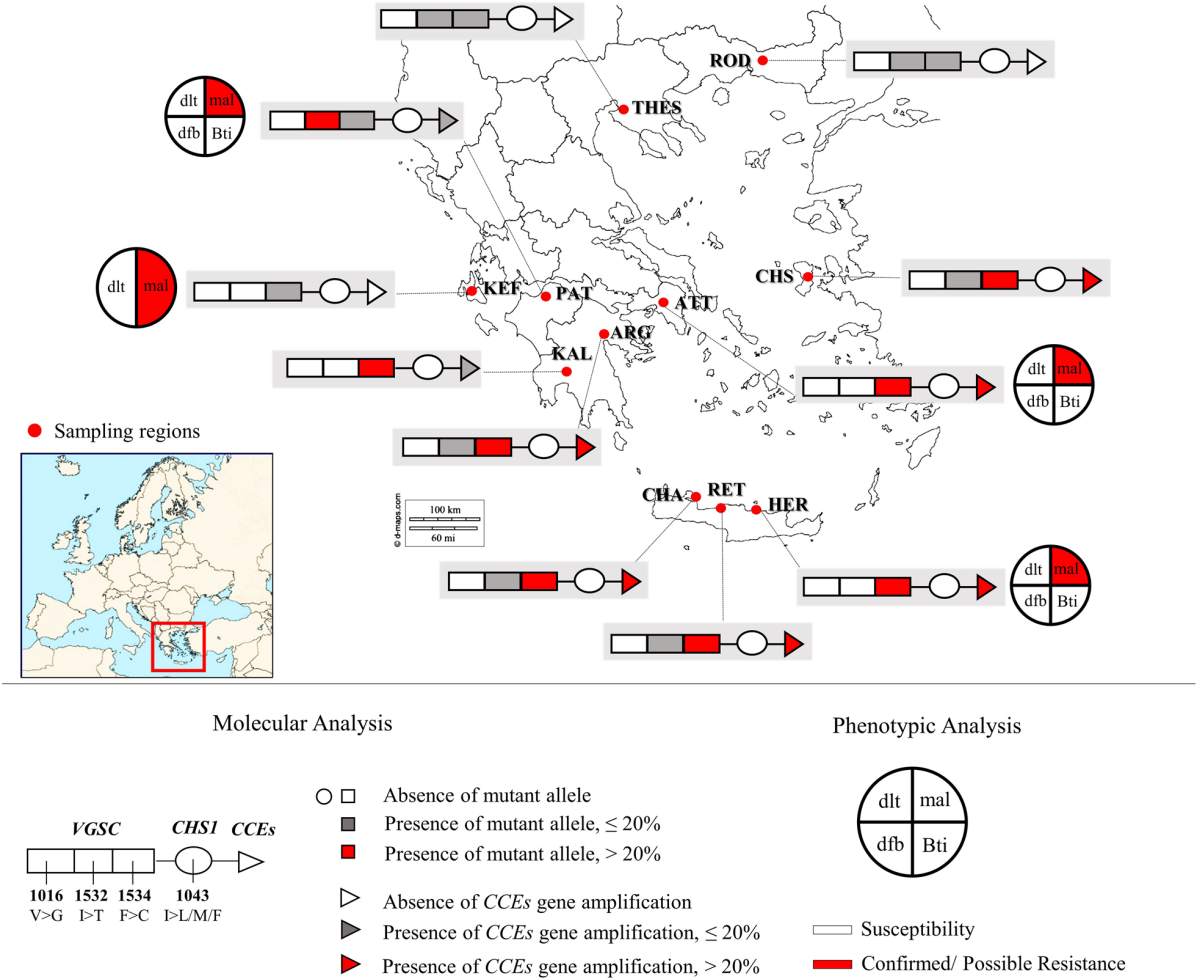
*Aedes albopictus* phenotypic and molecular analysis of insecticide resistance status, Greece. Sampling regions: ROD, Rodopi; THES, Thessaloniki; KEF, Kefalonia; PAT, Patras; ATT, Attica; CHS, Chios; KAL, Kalamata; ARG, Argolida; CHA, Chania; RET, Rethymno; HER, Heraklion. Molecular analysis includes the monitoring of: (i) mutations in the *VGSC* gene (substitutions in 3 loci: V1016G, I1532T and F1534C); (ii) mutations in the *CHS-1* gene (substitution I1043L/M/F); and (iii) the presence of multiple *CCEae3a* and *CCEae6a* gene copies. Phenotypic analysis in a subgroup of populations includes: (i) CDC bottle bioassays in adult mosquitoes against deltamethrin (dlt) and malathion (mal) and (ii) WHO larval bioassays against *Bti* and diflubenzuron (dfb)

Adult and immature stage *Ae. albopictus* mosquitoes were collected during the summer of 2017, 2018 and 2019, in a total of 19 urban and peri-urban localities in Greece, in the regions of Thessaloniki [33] and Rodopi (northern Greece), Attica and Argolida (central Greece), the Island of Chios (north-eastern Aegean Islands complex) [33], Patras and Kalamata (western Greece), the Island of Kefalonia (Ionian Islands complex) and Crete (Chania, Rethymno and Heraklion - southern Greece) (Fig.1, Table 1). The localities we selected to include in the analysis were chosen based on: their geographical location (in order to cover a large geographical area of Greece), the history of insecticide applications, previous insecticide resistance findings in other mosquito species and the availability of *Ae. albopictus* samples (mosquito collections/surveillance programmes).

**Table 1:** *Aedes* species composition per study site

Region	Locality	Collection date	*n*	*Ae. albopictus*	*Ae. cretinus*
Rodopi	Iasmos	August 2018	14	14	0
Thessaloniki	Thermi-Litsa^b^	September 2018	30	30	0
	Refugee camp Diavata^c^	June 2017	10^a^	10^a^	0
	Refugee camp Lagkadikia^c^	June 2017	18^a^	18^a^	0
Chios	Refugee camp Souda^c^	June 2017	23^a^	23^a^	0
Attica	Aigaleo	August-September 2018	24	24	0
	Filothei	August-September 2018	14	14	0
	Aghios Stefanos	October 2018	36	36	0
	Aghios Eleftherios	October 2018	24	24	0
Kefalonia	Paliki	November 2019	45	45	0
Patras	Rio	September 2019	33	33	0
Argolida	Aghia Triada	August-September 2018	8	8	0
	Kilada	September 2018	57	57	0
Kalamata	Town	August 2018	7	7	0
Chania	Town	September-October 2018	36	27	9
	Souda	August-October 2018	33	30	3
Rethymno	Town	August 2018	38	38	0
	Panormos	August-September 2018	18	18	0
Heraklion	Giofyro	June-July 2019	44	44	0

^a^Includes 10 eggs analyzed as a pool

^b^Thermi-Litsa is an organic farming locality

^c^Samples from Diavata, Lagkadikia and Souda refugee camps were collected and analysed in Fotakis et al. [33]

*Abbreviation*: *n*, total number of specimens analyzed per sampling region for species identification

Samples were collected every 2 weeks over a period of 1 or 2 months; a total of 3 to 7 collection events were conducted for each locality. Adult specimens were collected with mouth aspiration catches and CDC-light traps baited with dry ice. Larvae were sampled from natural and man-made/artificial containers with dipping collections and eggs were collected with oviposition traps (black plastic cups of 8 cm top diameter, 5 cm bottom diameter and 13 cm height; half covered with tap or rain water, with 2 wooden tongue depressors as oviposition substrate), baited with hay infusion and placed outdoors, amongst low vegetation, away from direct sunlight. Both larvae and eggs were collected from at least five different sites within each locality in order to avoid family bias and minimize the probability of including isofemale mosquitoes in the molecular analyses.

Following ovitrap collections, eggs were reared to adults in standard insectary conditions (temperature 27 ± 2 °C and relative humidity 70–80%), identified morphologically to species [34] and stored individually in absolute ethanol at 4 °C for the subsequent molecular analysis. A subgroup of eggs from the Aghios Stefanos locality (region of Attica; ovitrap collections from Aghios Stefanos were conducted in 2019, while samples analysed molecularly were collected in 2018), Kefalonia, Patras and Heraklion were reared to larvae or adults to use for susceptibility bioassays, as described below.

### Genomic DNA extraction and molecular identification of mosquito species

Genomic DNA (gDNA) was extracted from individual larvae or adult mosquitoes and from pools of eggs (10 eggs per pool per locality; 3 localities), using DNAzol reagent (Invitrogen, Carlsbad, CA, USA) according to manufacturer’s instructions.

Species identification was based on the PCR amplification (KAPA Taq PCR Kit; KAPA Biosystems) of the nuclear ribosomal gene spacer ITS2, following an assay that discriminates between *Ae. albopictus*, *Ae. cretinus* and *Ae. aegypti*, by generating PCR products of 509 bp, 385 bp and 324 bp in length, respectively, as described in Patsoula et al. [35]; the 25 µl PCR reaction contained 1 µl gDNA, 2.5 µl of 10× DNA polymerase buffer, 2 mM MgCl_2_, 0.4 µM of each primer (primers 5.8S and 28S [36]; Additional file 1: Table S1), 0.4 µM of dNTPs and 1.5 U of *Taq* polymerase. The applied thermal protocol was the following: initial denaturation at 94 °C for 5 min, 40 cycles × [denaturation at 94 °C for 1 min, primer annealing at 52 °C for 1 min, primer extension at 72 °C for 1 min] and a final extension step at 72 °C for 10 min. The PCR products were electrophoresed on a 1.5% w/v agarose gel containing ethidium bromide.

### Insecticide susceptibility bioassays

#### Larval bioassays

Following the WHO guidelines for laboratory and field testing of mosquito larvicides [37], we examined the susceptibility of *Ae. albopictus* populations against two larvicides; the bacterial larvicide *Bti* (VectoBac12AS, Valent BioSciences LLC, Illinois, USA; 1200 ITU (international toxic units)/mg; 11.61% w/v) and the insect growth regulator DFB (DU-DIM 15SC, Arysta LifeScience, Amsterdam, The Netherlands; 15% w/v). Both insecticides were diluted in distilled water. Bioassays were performed using *Ae. albopictus* third-early fourth-instar larvae (F_0_-F_1_ generation), reared under standard insectary conditions (temperature 27 ± 2 °C and relative humidity 70–80%). Fifteen to 20 larvae were placed in 99 ml water, to which 1 ml of the insecticide solution was added. Control bioassays contained 100 ml water. A range of 5 to 9 concentrations were tested for each insecticide (*Bti*: 0.008–0.500 mg/l; DFB: 0.0004–0.0200 mg/l) in order to define a mortality range between 10 and 95% and determine the LC_50_ and LC_95_ values. Three to 4 replicates were tested for each concentration. Larval mortality was recorded after the WHO recommended exposure time for each insecticide. Moribund larvae were counted as dead [37]. LC_50_ and LC_95_ values were estimated using the log-probit analysis Polo Plus 2.0 LeOra software (LeOra Software LLC, Parma, USA). Results were compared to the values reported for susceptible-laboratory *Ae. albopictus* strains in other studies [38–40].

#### Adult bioassays

Three to five day-old, non-blood fed female mosquitoes (F_1_–F_2_ generation) were subjected to insecticide susceptibility tests against deltamethrin and malathion, following the CDC bottle bioassay guidelines [41]. A Malaysian *Ae. albopictus* susceptible laboratory strain was included [42]. Both insecticides were purchased as technical grade material (PESTANAL® analytical standard; Sigma-Aldrich, Darmstadt, Germany). Insecticide stock solutions were prepared in acetone and Wheaton bottles were cleaned and coated as described in the CDC guidelines. The diagnostic dose of the insecticide under evaluation was used: deltamethrin at 10 µg/bottle and malathion at 50 µg/bottle. Tests were performed using 20–25 mosquitoes per bottle. Four insecticide treated replicate bottles and at least one control bottle (coated with acetone only) were used in each experiment set. The diagnostic time for both insecticides tested was 30 min [41]. Alive and dead mosquitoes in each bottle were recorded at time intervals of 5–15 min. The insecticide susceptibility status was determined by the mortality rate at the diagnostic time, according to CDC recommendations: 98–100% mortality at the diagnostic time indicates susceptibility; 80–97% suggests the possibility of resistance that requires further confirmation; and mortality < 80% denotes resistance. In cases where mortality (between 3–5%) was recorded in the control bottles at the 2 h timepoint, mortality data were corrected using Abbott’s formula.

### Genotyping of target site resistance mutations

#### Detection of knock-down resistance (kdr) mutations in the VGSC gene

The *VGSC* domain II was investigated for the presence of the V1016G mutation and domain III for mutations I1532T and F1534L/S/C *via* PCR and product sequencing.

The PCR (KAPA Taq PCR Kit) for domain II was carried out in 25 µl containing 1.5 µl of mixed gDNA extracted individually from 5–8 *Ae. albopictus* samples of the same locality, 2.5 µl of 10× DNA polymerase buffer, 0.4 µM of each primer (primers kdr2F and kdr2R; Additional file 1: Table S1), 0.4 µM of dNTPs and 1.5 U of *Taq* polymerase. The PCR thermal conditions were: initial denaturation at 95 °C for 5 min, 40 cycles × [denaturation at 94 °C for 30 s, primer annealing at 55 °C for 30 s, primer extension at 72 °C for 1 min] and a final extension step at 72 °C for 5 min. A small amount of the PCR products (5 µl) was electrophoresed on a 1% w/v agarose gel to verify the presence of the correct size amplicon (500 bp), and the remaining amount was purified using the Nucleospin PCR & Gel Clean-Up Kit (Macherey Nagel) and sequenced using the Sanger method (CeMIA S.A., Larissa, Greece) with primer kdr2F. Sequences were analysed using the sequence alignment editor BioEdit 7.2.5 (https://bioedit.software.informer.com/7.2/).

The PCR for domain III was carried out in 25 µl containing 1.5 µl of gDNA from *Ae. albopictus* individuals, 2.5 µl of 10× DNA polymerase buffer, 2 mM MgCl_2_, 0.3 µM of each primer (primersaegSCF7 and aegSCR7 [43]; Additional file 1: Table S1), 0.4 uM of dNTPs and 1.5 U of *Taq* polymerase. The thermal conditions of the PCR were: initial denaturation at 95 °C for 5 min, 40 cycles × [denaturation at 94 °C for 30 s, primer annealing at 57 °C for 30 s, primer extension at 72 °C for 1 min] and a final extension step at 72 °C for 5 min. The products were electrophoresed on a 1% w/v agarose gel and the specific 740 bp band was gel extracted and purified using the Nucleospin PCR & Gel Clean-Up Kit (Macherey Nagel, Dueren, Germany) and sequenced using the Sanger method (CeMIA S.A.) with primer aegSCR8. Sequences were analysed using the sequence alignment editor BioEdit 7.2.5.

#### Analysis of the CHS-1 1043 locus

Analysis of the *CHS-1* I1043 locus, to identify possible conserved DFB resistance mutations found in other species [29, 31], was performed in pools of mixed gDNA extracted individually from 5–8 *Ae. albopictus* samples of the same locality. Available *Ae. albopictus* DNA samples from other countries [28] were included in the analysis and genotyped individually. A 350-bp fragment of the *CHS-1* gene, spanning the 1043 locus (numbering based on *Musca domestica* genomic sequence) was amplified in a 25 µl PCR (KAPA Taq PCR Kit) containing 1.5 µl DNA, 2.5 µl of 10× DNA polymerase buffer, 0.4 µM of each primer (primers kkv F3 and kkv R3; Additional file 1: Table S1), 0.4 µM of dNTPs and 1.5 U of *Taq* polymerase. The thermal conditions were: initial denaturation at 95 °C for 5 min, 40 cycles × [denaturation at 94 °C for 30 s, primer annealing at 55 °C for 30 s, primer extension at 72 °C for 1 min] and final extension step at 72 °C for 10 min. A small amount of the PCR products was electrophoresed on a 1.5% w/v agarose gel containing ethidium bromide to verify amplification. The remaining amount of the PCR products was purified using the Nucleospin PCR & Gel Clean-Up Kit (Macherey Nagel) and sequenced using the Sanger method (CeMIA S.A.) with the kkv F3 primer. Sequences were analysed using the sequence alignment editor BioEdit 7.2.5.

### Metabolic resistance: detection of esterase gene amplification

*CCEae3a* and *CCEae6a* gene copy numbers were determined using quantitative real-time PCR on individual *Ae. albopictus* specimens. Amplification reactions at a 10 µl final volume were performed on a StepOnePlus Real-Time PCR System (Applied Biosystems, California, USA) containing 0.5 µl of gDNA, 0.2 µM of each primer (CCEae3aF, CCEae3aR, CCEae6aR and CCEEae6aF; Grigoraki et al. [28]; Additional file 1: Table S1) and SYBR Select Master Mix (Applied Biosystems, Thermo Fisher Scientific, California, USA). Histone 3 (GenBank: XM_019687528.2) was used as a reference gene for normalization (primers His3 TaqF and His3 TaqR; Additional file 1: Table S1). The thermal parameters were: 50 °C for 2 min; 95 °C for 2 min, and 40 cycles × [95 °C for 3 s, 60 °C for 30 s]. Melting curves were performed for reference and target genes to verify the presence of a unique specific PCR product, which was checked on a 1% w/v agarose gel. A no-template control was included to detect possible contamination. Two replicates per sample were included. *CCEae3a* and *CCEae6a* gene copy numbers were estimated relatively to a temephos susceptible *Αe. albopictus* laboratory strain from Greece.

## Results

### Molecular identification of mosquito species

A total of 482 individual mosquitoes (larvae and adults) and 30 eggs (in pools) were identified to species by PCR discrimination of the ITS2 genomic sequence length; 97.6% of the samples were identified as *Ae. albopictus*, while only 12 specimens, all from Chania, Crete, were identified as *Ae. cretinus* (corresponding to 17.4% of the Chania population) (Table 1). No *Ae. aegypti* mosquitoes were recorded.

### Insecticide susceptibility bioassays

#### Larval bioassays

*Aedes albopictus* populations from Aghios Stefanos-Attica (ATT), Patras and Heraklion, were tested for *Bti* resistance and found to be susceptible (Fig. 1). The calculated LC_50_ values were below 0.20 mg/l (corresponding to less than 0.240 ITU/ml) for all populations (Table 2), which is less than the LC_50_ values reported for susceptible *Ae. albopictus* laboratory strains in other studies. Indicatively, in Li et al. [39] and Su et al. [40], the reported LC_50_ values of the control susceptible strains were 0.036 mg/l and 0.044 mg/l, respectively (*Bti* formulation 7000 ITU/ mg).

**Table 2:** WHO bioassay mortalities for *Ae. albopictus* populations tested against *Bti*

*Bti*	Population	*n*	LC_50_ (95% CI)		LC_95_ (95% CI)		Slope ± SE	*χ*^2^	*df*
			mg/l	ITU/ml	mg/l	ITU/ml			
1200 ITU/mg	Patras	250	0.130 (0.080–0.171)	0.156 (0.096–0.205)	0.356 (0.250–0.994)	0.427 (0.300–1.193)	3.76 ± 0.45	113.3	23
	A. Stefanos- ATT	301	0.195 (0.156–0.235)	0.234 (0.187–0.282)	0.465 (0.360–0.736)	0.547 (0.432–0.883)	4.39 ± 0.41	88.59	28
	Heraklion	243	0.145 (0.113–0.179)	0.174 (0.136–0.215)	0.383 (0.276–0.874)	0.459 (0.331–1.049)	3.90 ± 0.49	75.46	23
7000 ITU/mg	Susceptible laboratory strains								
	Li et al. [39]		0.036 (0.028–0.047)	0.252 (0.196–0.329)					
	Su et al. [40]		0.044 (0.040–0.050)	0.308 (0.280–0.350)					

*Abbreviations*: ATT, Attica region; *n*, total number of larvae tested to a range of insecticide concentrations; LC_50_, lethal concentration (mg/l) that kills 50% of the population; LC_90_, lethal concentration (mg/l) that kills 95% of the population, CI, confidence intervals; ITU, international toxic units; *χ*^2^, Chi-square testing linearity of dose-mortality response with degrees of freedom (df); *df*, degrees of freedom

*Notes*: Log-dose probit-mortality data for larvicides tested against *Ae. albopictus* larvae. The results are compared to the susceptible laboratory *Ae. albopictus* control strains of other studies [39, 40]

All three populations were also tested for diflubenzuron resistance and showed mortality of 100% in DFB doses below 0.02 mg/l. This is remarkably lower than the recommended field doses (DU-DIM 15SC: 0.32–0.63 mg/l), the recommended WHO dosage of DFB in potable water containers (0.25 mg/l) [44], as well as the emergence inhibition dose (EI_50_ of 0.376 mg/l) previously reported for susceptible *Ae. albopictus* field strains [38].

#### Adult bioassays

*Aedes albopictus* populations from Aghios Stefanos-Attica (ATT), Kefalonia, Patras and Heraklion were susceptible to deltamethrin (Fig. 1), as the mortality recorded at the 30 min diagnostic time was 100% (Table 3, Additional file 2: Figure S1). On the other hand, populations from Aghios Stefanos-Attica (ATT), Kefalonia and Heraklion, were resistant to malathion, displaying approximately 55% mortality at the diagnostic time (30 min), while the Patras population had a mortality of 91.4%, indicating possible resistance (Table 3, Additional file 2: Figure S1).

**Table 3:** CDC bottle bioassay mortalities for *Ae. albopictus* populations tested against deltamethrin (pyrethroid) and malathion (OP)

Population	Deltamethrin 10 µg/ml			Malathion 50 µg/ml		
	*n*_*1*_	Mortality (%) (95% CI)	Status	*n*_*2*_	Mortality (%) (95% CI)	Status
A. Stefanos-ATT	75	100 (–)	S	79	55.70 (28.55–82.85)	R
Patras	70	100 (–)	S	83	91.4 (83.10–99.70)	R^a^
Kefalonia	88	100 (–)	S	83	55.6 (15.15–96.05)	R
Heraklion	78	100 (–)	S	78	55.3 (33.70–76.90)	R
Susceptible LC	70	100 (–)	S	76	100 (–)	S

^a^Possibility of resistance

*Notes*: Mortality percentages correspond to the discriminating exposure time DT=30 min for both insecticides [40]. The average values of four insecticide treated replicate bottles are presented. *n*_*1*_ and *n*_*2*_ refer to the total number of female mosquitoes tested against deltamethrin and malathion, respectively

*Abbreviations*: ATT, Attica region; CI, confidence intervals; LC, laboratory colony; R, resistance; S, susceptibility (according to WHO recommendations for CDC bottle bioassay guidelines, 2010)

### Genotyping of target site resistance mutations

#### VGSC: kdr mutations at positions V1016, I1532 and F1534

As the V1016G *kdr* mutation has never been recorded in Greece, pooled gDNA was used as a template for *VGSC* domain II amplification. Genotyping of the *VGSC* domain II was performed for a total of 323 larvae/adult mosquitoes (in 2–10 pools per region, depending on the number of specimens analysed per locality; 5–8 individuals per locality per pool) and 20 eggs (in pools of 10 per locality). The small number of specimens included per pool would enable the detection of any resistance allele upon sequencing. The wild type allele V1016 (codon GTA) was recorded in all cases (Fig. 1, Table 4).

**Table 4:** Genotype and allele frequencies (%) of *VGSC* domain II locus V1016 and domain III loci I1532 and F1534

Region	Year	V1016G		I1532T						F1534C				
		*N*_*1*_	% allele freq	*N*_*2*_	Genotype			% allele freq		Genotype			% allele freq	
			(V)		II	IT	TT	(I)	(T)	FF	FC	CC	(F)	(C)
Rodopi	2018	13	100	12	11	1	0	95.8	4.2	8	4	0	83.3	16.7
Thessaloniki														
Diavata, Lagkadikia^b^	2017	18^a^	100	8	8	0	0	100	0	7	1	0	93.8	6.2
Thermi-Litsa	2018	30	100	30	28	2	0	96.7	3.3	26	4	0	93.3	6.7
Chios^b^	2017	22^a^	100	11	11	0	0	100	0	6	4	1	72.7	27.3
Attica	2018	59	100	52	52	0	0	100	0	12	9	31	31.7	68.3
Kefalonia	2019	45	100	41	41	0	0	100	0	37	1	3	91.5	8.5
Patras	2019	33	100	33	20	11	2	77.3	22.7	28	5	0	92.4	7.6
Argolida	2018	21	100	21	19	2	0	95.2	4.8	10	3	8	54.8	45.2
Kalamata	2018	7	100	6	6	0	0	100	0	2	3	1	58.3	41.7
Rethymno	2018	20	100	30	29	1	0	98.3	1.7	10	11	9	51.7	48.3
Chania	2018	31	100	31	27	4	0	93.5	6.5	15	14	2	71.0	29.0
Heraklion	2019	44	100	44	44	0	0	100	0	15	19	10	55.7	44.3

^a^Includes 10 eggs analysed in a pool. Results are presented cumulatively for the different sampling localities in each region, except for Thessaloniki ^b^Samples from Diavata and Lagkadikia refugee camps (Thessaloniki) and Chios were analysed in Fotakis et al. [33]

*Abbreviations*: *N*_*1*_ and *N*_*2*_, total number of genotyped specimens for *VGSC* domain II and domain III respectively, per sampling region; V, 1016V susceptible allele. Genotypes for V*GSC* locus 1016 are not given as all samples were wild-type 1016V/1016V; I, 1532I susceptible allele; T, 1532T mutant allele; II, 1532I/1532I homozygous; IT, 1532I/1532T heterozygote; TT, 1532T/1532T homozygous mutant; F, 1534F susceptible allele; C, 1534C mutant allele; FF, 1534F/1534F homozygous; FC, 1534F/1534C heterozygote; CC, 1534C/1534C homozygous mutant

For the detection of mutations I1532T and F1534C/L/S in the *VGSC* domain III, 319 individuals were genotyped. For the first time in Greece, the I1532T mutation was detected in 6 out of the 11 surveyed regions. Particularly, 23 genotyped specimens from Rodopi, Thessaloniki, Patras, Argolida, Rethymno and Chania were found to have this substitution, mostly in heterozygosis (genotype 1532I/1532T). In the majority of the above-mentioned regions, the mutant allele 1532T frequency was low, varying from 1.7 to 6.5% (Fig. 1). The highest frequency was observed in Patras (22.7%; the only 2 homozygotes 1532T/1532T were reported there) (Table 4).

The F1534C mutation was found in all regions. The regions with the highest 1534C allele frequency were Attica (68.3%), Argolida (45.2%), Rethymno (48.3%), Heraklion (44.3%) and Chania (29%), all located in central and southern Greece (we excluded Kalamata results due to the small number of specimens collected and analysed). In contrast, regions from northern Greece (Rodopi and Thessaloniki) and western Greece (Patras and Kefalonia) displayed lower 1534C allele frequencies, ranging between 6.6 and 16.7% (Fig. 1). The F1534C mutation appeared mainly in heterozygosis, with the exception of Attica, where more than half of the genotyped specimens were homozygous for the mutation (genotype 1543C/1534C) (Table 4). Only two individuals (sampled from Argolida and Patra) harboured both mutations, I1532T and F1534C, in heterozygosis (genotype 1532I/1532T; 1534F/1534C).

#### Chitin synthase (CHS-1): mutations at position I1043

The *CHS-1* genomic sequence of 325 *Ae. albopictus* mosquitoes (in 2–10 pools per region, depending on the number of specimens analysed per locality; 5–8 individuals per locality per pool) and a total of 20 eggs (in pools of 10 per locality) were analysed for the presence of either of the three mutations I1043L/M/F, previously linked to DFB resistance. No mutations were detected (Fig.1, Additional file 1: Table S2). Likewise, no mutations were recorded in the 178 genotyped samples from USA, Brazil, Belize, Gabon, Switzerland, Taiwan, France, Mexico, China, Sri Lanka, Australia, Japan, Lebanon and Bangladesh (Additional file 1: Table S3).

### Esterase gene amplification

*CCEae3a* and *CCEae6a* amplification associated with temephos resistance was recorded in 8 out of the 11 surveyed regions in Greece (Fig. 1). Two types of amplification were found: a *CCEae3a* amplicon and a *CCEae3a* - *CCEae6a* co-amplicon.

**Fig. 2:**
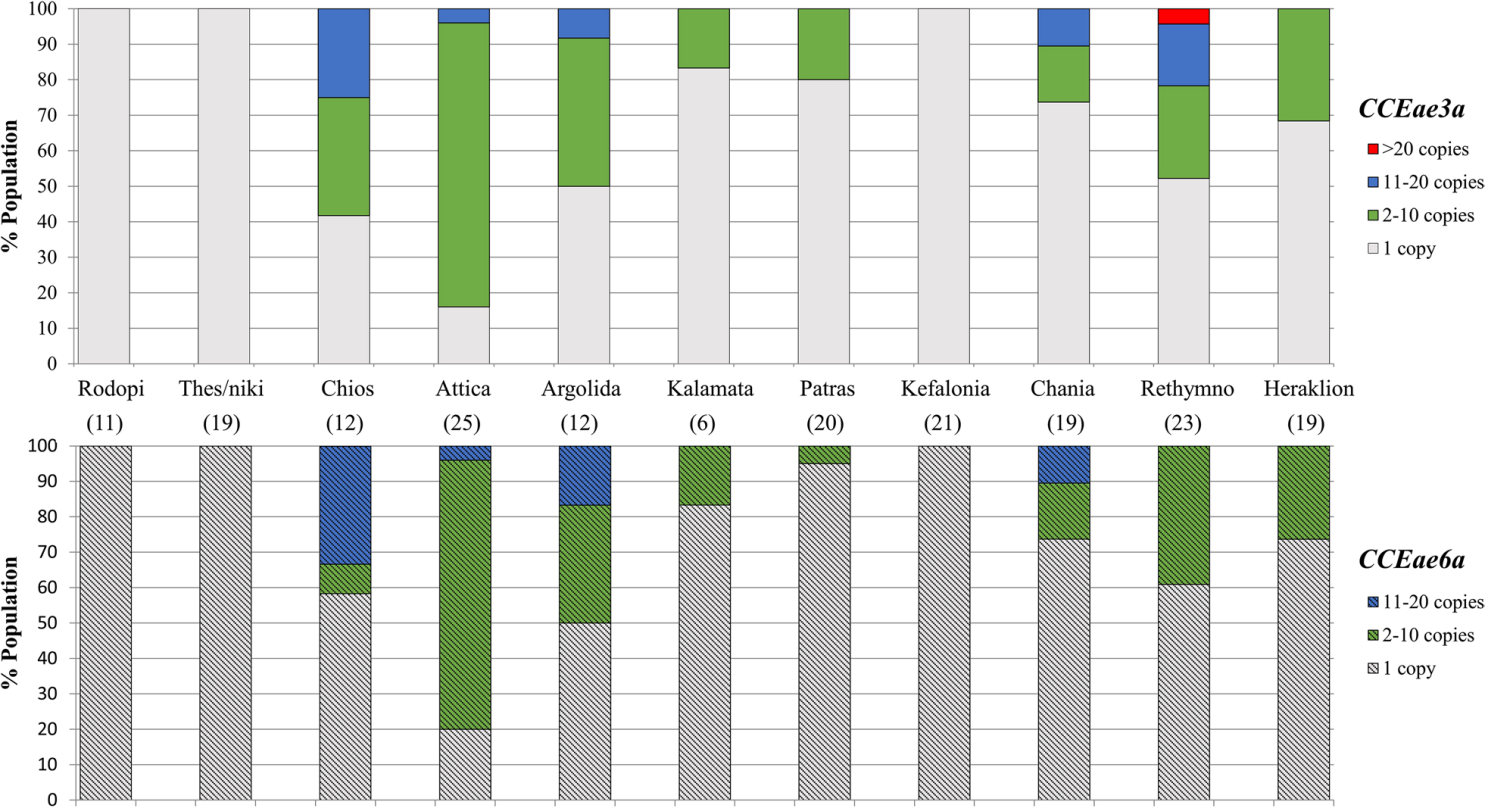
Distribution of the relative *CCEae3a* and *CCEae6a* gene copy numbers in *Aedes albopictus* per sampling region. Solid bars (upper panel) refer to relative *CCEae3a* and patterned bars (lower panel) to relative *CCEae6a* gene copy numbers. Numbers in parentheses correspond to the number of specimens commonly analysed for both *CCEs* gene amplification with quantitative PCR. Results of each region presented are cumulative of different localities

Amplified esterase genes were detected in specimens from Chios, Argolida, Patra, Kalamata, Attica, Chania, Rethymno and Heraklion. The reported frequency of the *CCeae3a* amplification, in the eight locations, ranged from 16.6 to 84% and that of *CCEae3a-CCEae6a* co-amplification from 5 to 80%. The majority of samples with amplified esterases harboured between 2–10 gene copies. Individuals with more than 10 (11–20) and more than 20 gene copies (in the case of one individual from Rethymno) were also recorded. Attica was the region with the highest percentage of individuals carrying ≥ 2 copies of either *CCEae3a* or *CCEae6a* gene (84% and 80%, respectively) and Chios of individuals with > 10 copies (25% and 33.3%, respectively) (Fig. 2).

No carboxylesterase gene amplification was detected in Rodopi, Thessaloniki and Kefalonia populations.

## Discussion

This study represents an extended investigation of the insecticide resistance status of *Ae. albopictus* in Greece. Entomological monitoring revealed the dominant presence of *Ae. albopictus* over other *Aedes* container-breeding species and confirmed the species’ widespread distribution in the country [12, 45]. The discrimination of *Ae. albopictus* from morphologically similar *Stegomyia* species using the nuclear ITS2 as a molecular marker [35] is of high importance in order to distinguish this species from the primary arbovirus vector *Ae. aegypti* [46] and the non-vector *Ae. cretinus* [47]. The latter, although considered a native mosquito species in Greece [48] extensively reported in the past in Attica and Crete [45], was detected in our study only in Chania, Crete corresponding to an overall frequency of 2.4%. The absence/very low *Ae. cretinus* frequency in the sampled urban and peri- urban settings may be correlated to the competitive and highly adaptable behavior displayed by *Ae. albopictus* [49].

The *Ae. albopictus* WHO and CDC bioassays showed full susceptibility of the tested populations against deltamethrin and the larvicides DFB and *Bti*, suggesting their current suitability for inclusion in vector control programmes in Greece. On the contrary, mortality levels < 90% were recorded in the malathion assays indicating resistance against this OP insecticide. Although malathion and OPs are not currently used in Greece for mosquito control, OPs are still used in agriculture and thus possibly maintain a selection pressure also against *Ae. albopictus*. Alternatively, the fitness cost of the *CCE* amplification may be negligible, to allow a decrease in OP resistance. Nevertheless, the observed resistance raises some level of concern, regarding the potential need for re-introducing OP insecticides, in case of public health emergency.

Genotyping of the *VGSC* gene for the detection of mutations associated with pyrethroid resistance revealed a widespread distribution of the *kdr* mutation F1534C across the country, following initial reports of the mutation in Greece [50, 51]. The recorded 1534C allele frequencies were above 40% in populations from southern and central Greece, peaking in Attica (68.3%). Previous studies also report relatively high mutation frequencies (reaching 66%) in populations sampled from the Attica region [50, 51]. To date, *kdr* F1534C has also been recorded in Brazil [52], Singapore, Vietnam [53] and China [54], while other substitutions in the same genomic locus, F1534L and F1534S, also associated with pyrethroid resistance, have been identified in the USA, China, Vietnam and Italy [50, 53–55].

The involvement of both F1534L and F1534S mutations in resistance to type I pyrethoids was recently further supported by *in vitro* functional characterization data [24]. The F1534L substitution was shown to confer similar levels of insensitivity to the previously characterized F1534C, while F1534S seemed to have an even bigger effect. Thus, monitoring the presence of these mutations in countries, like Greece, where type I pyrethroids are used for vector control is of great importance.

The F1534C mutation in the homozygous state has been associated with resistance to permethrin [23] in *Aedes* mosquitoes. In the analysed populations, F1534C was observed mainly in heterozygosis, potentially accounting for low resistance levels, due to the recessive nature of the mutant 1534C allele [56]. Nevertheless, the recorded mutation frequencies should not be undermined, as a notable raise of insecticidal pressure may lead to rapid increase in mutation selection potentially hampering the effectiveness of permethrin based applications for *Ae. albopictus* control. Additionally, the investigation of P450s-mediated pyrethroid detoxification, which in combination with target-site mutations has been shown to confer operationally significant resistance levels [57], would be critical complementary evidence facilitating the development of efficient control programmes.

Another mutation in the *VGSC* gene, I1532T, was found in Greece at low frequencies (< 10%) in several surveyed regions with the exception of Patras (western Greece) where the mutant allele reached a frequency of 22.7%. This mutation has also been reported at considerable frequencies in *Ae. albopictus* populations from Italy [50, 51], Albania [51] and China [54]. Although Kasai et al. [53] demonstrated a lack of association between this mutation and resistance to pyrethroids, a recent study revealed that VGSC channels carrying this mutation have reduced sensitivity to type I pyrethoids, notably at similar levels to channels harbouring the F1534S mutation [24]. Therefore, it is important to monitor the presence and distribution of this mutation in field populations. We also identified in our samples two individuals carrying both the I1532T and F1534C mutations. In some cases, mutation co-occurrence can have an additive or even synergistic effect resulting in very high levels of resistance [22]. Although the co-occurrence of I1532T + F1534C has not yet been functionally characterized, the co-occurrence of I1532T with F1534S or F1534L was recently shown to have a similar effect as the single mutants in conferring type I pyrethroid insensitivity [24].

The V1016G *kdr* mutation, correlated with “stronger” resistance (than F1534C) to type I and type II pyrethroids [23, 53] was not detected in any of the analysed samples from Greece. However, it was recently recorded in *Ae. albopictus* populations from Hanoi (Vietnam), in Beijing (China) and across Italy [51, 53, 58]. The systematic monitoring of this mutation in *Ae. albopictus* populations from Greece and elsewhere is strongly recommended, given the primary role of pyrethroids in *Aedes* mosquito control worldwide [18] and the possibility of low mutation frequencies and/or focal mutation distribution going undetected.

Resistance to DFB was assessed molecularly in specimens from all 11 surveyed regions in Greece through monitoring of the substitutions I1043L/M/F in the *CHS-1* gene, associated with high resistance levels in *Cx. pipiens* mosquitoes [29–31]. No *CHS-1* mutations were recorded, a result in line with the respective bioassay outcome, indicating the effectiveness of DFB for *Ae. albopictus* control in the study regions. Likewise, all samples analysed from France, Switzerland, Lebanon, Belize, Gabon, Taiwan, Sri Lanka, China, Japan, Australia, Bangladesh, USA, Brazil and Mexico were homozygous for the wild type (susceptible) I1043 allele, indicating that this resistance mechanism is likely not yet present in *Ae. albopictus* worldwide. However, the possibility of a focal DFB resistance pattern, in line with *Cx. pipiens* findings [30] cannot be excluded, emphasizing the need for incorporating systematic monitoring of DFB resistance mutations in integrated *Ae. albopictus* surveillance programmes.

Carboxylesterase *CCEae3a* and *CCEae6a* gene amplifications linked to temephos resistance [27, 28] were detected in 8 out of the 11 surveyed regions. Almost 33% of the total *Ae. albopictus* specimens analysed, the majority of which were sampled from central and southern Greece, carried more than 1 copy of *CCEae3a* or both *CCEae3a-CCEae6a* genes indicating elevated temephos detoxification.

Interestingly out of the 156 *Ae. albopictus* samples commonly analysed for the detection of the F1534C *kdr* mutation and *CCEae3a* amplification, 25% were found to harbor both mutations, denoting a potential risk for multi-resistance against pyrethroid and organophosphate insecticides.

The observed resistance against malathion may be associated with the high occurrence of amplified *CCEs* in the analysed populations. *CCEae3a* and *CCEae6a* gene amplification was originally associated with temephos resistance in *Ae. albopictus* mosquitoes [27], yet Marcombe et al. [59] also correlated the presence of multiple *CCEae3a* gene copies with malathion resistance in *Ae. aegypti* mosquitoes. Thus, it is possible that the detected *CCEs* amplifications in the Greek *Ae. albopictus* populations are responsible for the resistance levels recorded in the malathion bioassays, by conferring cross-resistance across additional OPs.

Evident differences were noted in the frequency of the F1534C *kdr* mutation, between populations from Thessaloniki (northern Greece; mutant F1534C allele frequency < 7%) and sampling locations in central and southern Greece (mutant F1534C allele frequencies > 29%). Although we cannot exclude the scenario of the passive transportation of populations harbouring insecticide resistance mutations, these differences may also partially reflect the imposed insecticidal pressures. Similarly, in Thessaloniki *CCEs* gene amplification was not detected in any sample, while 26–84% of the Attica, Argolida, Chania, Rethymno and Heraklion populations had more than one *CCEae3a* gene copy. The surveyed regions of central and southern Greece have intense agricultural activities, where pyrethroids and OPs have been used extensively for pest control over the years, possibly contaminating nearby *Ae. albopictus* breeding sites and leading to the selection of the respective resistance mutations [60]. On the contrary, in Thessaloniki the majority of the specimens were sampled from an organic farm (Litsa-Thermi) where no insecticides have been applied over the last ten years possibly explaining the low *kdr* mutation frequency and the absence of *CCEs* amplification.

The limited number of approved insecticides for public health purposes and the worldwide data on *Ae. albopictus* insecticide resistance generated to date raise concerns regarding the effectiveness and suitability of the current vector control interventions. Our findings significantly enrich the available information on *Ae. albopictus* insecticide resistance status. In a context of evidence-based vector control programmes, regular investigation of the species composition, population dynamics and systematic resistance monitoring encompassing both bioassays and molecular diagnostics, providing highly complementary yet not redundant information, are critical components for the development of resistance management strategies.

## Conclusions

This study delineates the susceptibility profile of *Ae. albopictus* populations from Greece against commonly used insecticides. Our findings suggest the suitability of the larvicides DFB and *Bti* and the current effectiveness of pyrethroids for *Ae. albopictus* control. However, the presence of high *kdr* mutation frequencies raises concerns, given the dominant role of pyrethroids in mosquito control and the few alternative synthetic compounds available for public health purposes. The OPs malathion and temephos appear unreliable alternative insecticides for possible future re-introduction in mosquito control. Systematic monitoring of the insecticide resistance traits is imperative for the development of resistance management programmes ensuring the sustainability of the current chemical control tools.

## Supplementary information

**Additional file 1: Table S1.** Primers used in this study for regular and real-time PCR. **Table S2.** Genotype and allele frequencies of the C*HS-1* locus 1043, Greece. **Table S3**. Genotype and allele frequencies of the C*HS-1* locus 1043, other countries.

**Additional file 2: Figure S1.** CDC bioassay mortality percentages corresponding to exposure time against malathion and deltamethrin for *Aedes albopictus* populations from Greece.

## Data Availability

All data generated or analysed during this study are included in this published article and its additional files.

## Abbreviations

*Bti*: *Bacillus thuringiensis* var. *israelensis*
CCE: carxoxylesterase
(E)CDC: (European) Centre for Disease Control
CHIKV: chikungunya virus
*CHS-1*: chitin synthase-1
CI: confidence interval
CYP: cytochrome P450 monooxygenase
DDT: dichlorodiphenyl-trichloroethane
DENV: dengue virus
*df*: degrees of freedom
DFB: diflubenzuron
EI: emergence inhibition
GCN: gene copy number
gDNA: genomic DNA
IGR: insect growth regulator
ITS2: internal transcribed spacer 2
ITU: international toxic units
LC: lethal concentration
MBDs: mosquito-borne diseases
OP: organophosphate
PCR: polymerase chain reaction
SD: standard deviation
VBDs: vector-borne diseases
*VGSC*: voltage-gated sodium channel
WHO: World Health Organization
WNV: West Nile virus
