# Supplementary material for: Bioassay and molecular monitoring of insecticide resistance status in Aedes albopictus populations from Greece, to support evidence-based vector control
Source: Parasit Vectors. 2020 Jun 29;13:328. doi: 10.1186/s13071-020-04204-0 (PMC7325023; doi:10.1186/s13071-020-04204-0)
Supplement: Supplementary file 1 — Additional file 1: Table S1. Primers used in this study for regular and real-time PCR. Table S2. Genotype and allele frequencies of the CHS-1 locus 1043, Greece. Table S3. Genotype and allele frequencies of the CHS-1 locus 1043, other countries. [file 13071_2020_4204_MOESM1_ESM.docx]

**Additional file 1: Table S1.** Primers used in this study for regular and real-time (*) PCR.

| **Primer Name** | **Primer Sequence** | **Reference** |
| --- | --- | --- |
| 5.8S | 5’ TGTGAACTGCAGGACACATG 3’ | [59] |
| 28S | 5’ ATGCTTAAATTTAGGGGGTA 3’ | [59] |
| kkv F3 | 5' TCGGAAGTCCTTCGGCTTATTC 3' | - |
| kkv R3 | 5' TGGATACTTCAATGGAACCTTCC 3' | - |
| kdr2 F | 5’ TTCACCGACTTCATGCACTC 3’ | - |
| kdr2 R | 5’ CGCAATCTGGCTTGTTAACTT 3’ | - |
| aegSCF7 | 5’GAGAACTCGCCGATGAACTT 3’ | [60] |
| aegSCR7 | 5’GACGACGAAATCGAACAGGT 3’ | [60] |
| aegSCR8 | 5’ AGCTTTCAGCGGCTTCTTC 3’ | [60] |
| His3 Taq F* | 5’ CCCAAGATTTCAAGACCGATCT 3’ | - |
| His3 Taq R* | 5’ GGTAGGCTTCACTGGCTTCCT 3’ | - |
| CCEae3a F* | 5’ AGAGTGCGTTACGGATCAAG 3’ | [28] |
| CCEae3a R* | 5’ TAGCCTCATTGCTGGTTAGC 3’ | [28] |
| CCEae6a F* | 5‘ CAGCATGTCCTCGTTAAAGC 3' | [28] |
| CCEae6a R* | 5‘ GACAACACACTTCCCTACCG 3' | [28] |

**Table S2:** Genotype and allele frequencies (%) of the C*HS-1* locus 1043, Greece.

| **Region** |  |  | ***N*** | ***CHS-1* 1043** | | | |
| --- | --- | --- | --- | --- | --- | --- | --- |
|  | **Collection**  **Year** |  |  | **Genotype** |  | **% allele freq** | |
|  |  |  |  | **I/I** |  | **(I)** | **(M) / (L) / (F)** |
| Rodopi | 2018 |  | 13 | 13 |  | 100 | 0 |
| Thessaloniki^¥^ | 2017, 2018 |  | 68* | 68* |  | 100 | 0 |
| Chios^¥^ | 2017 |  | 12 | 12 |  | 100 | 0 |
| Attica | 2018 |  | 52 | 52 |  | 100 | 0 |
| Kefalonia | 2019 |  | 45 | 8 |  | 100 | 0 |
| Patras | 2019 |  | 33 | 33 |  | 100 | 0 |
| Argolida | 2018 |  | 22 | 22 |  | 100 | 0 |
| Kalamata | 2018 |  | 7 | 7 |  | 100 | 0 |
| Chania | 2018 |  | 25 | 25 |  | 100 | 0 |
| Rethymno  Heraklion | 2018  2019 |  | 24  44 | 24  44 |  | 100  100 | 0  0 |

*N*: total number of specimens genotyped in pools per sampling region. Results are cumulatively of all localities per region, as only the wild type allele (1043I) was detected. ¥: Samples from Diavata, Lagkadikia (Thessaloniki, 2017) and Souda (Chios) refugee camps were analysed for Fotakis *et al.,* 2019 [33]. I: 1043I wild-type susceptible allele, L/M/F: 1043L/M/F mutant alleles; II: 1043I/1043I homozygous susceptible. *: 20 eggs analysed in pools (10 eggs per pool per locality) included.

**Table S3:** Genotype and allele frequencies (%) of the C*HS-1* locus 1043, other countries.

| **Country** | **Region** |  |  | ***N*** | ***CHS-1* 1043** | | | |
| --- | --- | --- | --- | --- | --- | --- | --- | --- |
|  |  | **Collection**  **Year** |  |  | **Genotype** |  | **% allele freq** | |
|  |  |  |  |  | **I/I** |  | **(I)** | **(M) / (L) / (F)** |
| USA | Florida, Atlanta | 2014, 2015 |  | 24 | 24 |  | 100 | 0 |
| Brazil | Rio de Janeiro | 2015 |  | 12 | 12 |  | 100 | 0 |
| Belize | Orange Walk town | 2015 |  | 10 | 10 |  | 100 | 0 |
| Gabon | Franceville, Cocobeach | 2015 |  | 15 | 15 |  | 100 | 0 |
| Switzerland | Ticino | 2012, 2013 |  | 13 | 13 |  | 100 | 0 |
| Taiwan | Taipei | 2014 |  | 12 | 12 |  | 100 | 0 |
| France | Montpellier | 2015 |  | 10 | 10 |  | 100 | 0 |
| Mexico | Tapachula | 2015 |  | 12 | 12 |  | 100 | 0 |
| China | Beijing | 2015 |  | 12 | 12 |  | 100 | 0 |
| Sri Lanka | Peradeniya | 2014 |  | 14 | 14 |  | 100 | 0 |
| Australia | Hammond | 2012 |  | 14 | 14 |  | 100 | 0 |
| Japan | Tokyo | 2015 |  | 12 | 12 |  | 100 | 0 |
| Lebanon | Beirut | 2015 |  | 10 | 10 |  | 100 | 0 |
| Bangladesh | Panchagarh | 2015 |  | 8 | 8 |  | 100 | 0 |

*N*: total number of adult specimens genotyped indivivually per country. I: 1043I wild-type susceptible allele, L/M/F: 1043L/M/F mutant alleles; II: 1043I/1043I homozygous susceptible.
