# Supplementary material for: Bioassay and molecular monitoring of insecticide resistance status in Aedes albopictus populations from Greece, to support evidence-based vector control
Source: Parasit Vectors. 2020 Jun 29;13:328. doi: 10.1186/s13071-020-04204-0 (PMC7325023; doi:10.1186/s13071-020-04204-0)
Supplement: Supplementary file 2 — Additional file 2: Figure S1. CDC bioassay mortality percentages corresponding to exposure time against malathion and deltamethrin for Aedes albopictus populations from Greece. [file 13071_2020_4204_MOESM2_ESM.docx]

**Additional file 2: Figure S1.** CDC bioassay mortality percentages corresponding to exposure time (minutes) against malathion and deltamethrin, for *Aedes albopictus* populations from Greece.

Percentages presented are cumulative of 4 insecticide treated replicate bottles. Black dotted line indicates the 98% cut-off for insecticide resistance (CDC guidelines; [40]).
